# MicroRNA-7-5p mediates the signaling of hepatocyte growth factor to suppress oncogenes in the MCF-10A mammary epithelial cell

**DOI:** 10.1038/s41598-017-15846-z

**Published:** 2017-11-13

**Authors:** Dawoon Jeong, Juyeon Ham, Sungbin Park, Seungyeon Lee, Hyunkyung Lee, Han-Sung Kang, Sun Jung Kim

**Affiliations:** 1 Department of Life Science, Dongguk University-Seoul, Goyang, Republic of Korea; 2 Research Institute and Hospital, National Cancer Center, Goyang, Republic of Korea

## Abstract

MicroRNA-7 (miR-7) is a non-coding RNA of 23-nucleotides that has been shown to act as a tumor suppressor in various cancers including breast cancer. Although there have been copious studies on the action mechanisms of miR-7, little is known about how the miR is controlled in the mammary cell. In this study, we performed a genome-wide expression analysis in miR-7-transfected MCF-10A breast cell line to explore the upstream regulators of miR-7. Analysis of the dysregulated target gene pool predicted hepatocyte growth factor (HGF) as the most plausible upstream regulator of miR-7. MiR-7 was upregulated in MCF-10A cells by HGF, and subsequently downregulated upon treatment with siRNA against HGF. However, the expression of HGF did not significantly change through either an upregulation or downregulation of miR-7 expression, suggesting that HGF acts upstream of miR-7. In addition, the target genes of miR-7, such as EGFR, KLF4, FAK, PAK1 and SET8, which are all known oncogenes, were downregulated in HGF-treated MCF-10A; in contrast, knocking down HGF recovered their expression. These results indicate that miR-7 mediates the activity of HGF to suppress oncogenic proteins, which inhibits the development of normal cells, at least MCF-10A, into cancerous cells.

## Introduction

MicroRNAs (miRs) are small non-coding RNAs that are approximately 20–25 nucleotides long, with over 2500 mature forms identified in the human genome^1^. MiRs have the capacity to regulate numerous target genes at a post-transcriptional level and sequence-specific manner by binding to the 3′-untranslated regions of target mRNAs^2^. MiRs are involved in diverse biological process including cell development, proliferation and carcinogenesis, and have thus emerged as potential diagnostic markers and therapeutic targets for various cancers^3^. MiR-7 is derived from three primary transcripts, namely pri-miR-7-1, pri-miR-7-2 and pri-miR-7-3, which are all encoded from different genomic loci, 9q21, 15q26 and 19q13, respectively^4^. Downregulation of miR-7 has been revealed in various cancer types including breast, gastric and lung cancer^5–7^, with lower miR-7 transcript levels associated with poorer prognosis in cancer patients. MiR-7 shows tumor suppressive activity by targeting many oncogenes such as EGFR, FAK, PAK1, KLF4 and SET8 in breast and other cancers^8,9^. In line with its cancer suppressive activity, the overexpression of miR-7 mimics showed a decreased cell growth and invasion, while those overexpressing miR-7-antisense showed the opposite effects^10,11^.

Hepatocyte growth factor (HGF) stimulates cell proliferation and differentiation of various cell types^12^. For example, the differentiation of bone mesenchymal stem cells (BMSCs) can be induced through HGF-activated NF-кB signaling^13^. In mouse and human mammary glands, HGF acts as a morphogenic factor in mammary epithelial cells, generating tubular branching and well-constructed lumina during differentiation and development^14^. Upregulation of HGF has been frequently observed in various cancers wherein higher expression of HGF is associated with poorer prognosis in cancer patients^15^. In contrast, the tumor suppressive functions of HGF in cancer is sparsely known. A recent study has indicated that an HGF-regulated tyrosine kinase substrate harboring tumor suppressive activity is regulated by HGF stimulation^16^. HGF binds to its only receptor, Met, and thereafter initiates a series of signaling pathways that include the activation of Erk1/2 and Wnt/β-catenin signaling, as well as the AKT pathway^17^.

A few miRs have been identified to mediate the activity of HGF. One example is miR-124, which is downregulated in HGF-treated mesenchymal stem cells (MSC)^18^. MiR-124 downregulates Wnt/β-catenin signaling by targeting FZD4 and LRP6, thus suppressing the chemotactic migration of rat MSCs toward HGF. MiR-211 and miR-26b are also regulated by HGF in MSC, but are instead upregulated by HGF to activate PI3K/Akt signaling through targeting PTEN^19^. In renal cell carcinoma, miR-199a-3p inhibited HGF/c-Met signaling, which included the STAT3, mTOR and ERK1/2 pathways; however, whether miR-199a-3p itself is regulated by HGF has not yet been elucidated^20^.

Albeit the battery of experimental results that investigate the biological functions of miR-7 and HGF in cancer cells, there are presently no reported association between them. In this study, we explored the target genes of miR-7, which then identified HGF as an upstream regulator of the miR. This observation was then supported by the downregulation of miR-7 after inhibition of HGF using siRNA in MCF-10A mammary cells. In addition, the expression profile of miR-7 target genes was examined after the upregulation or downregulation of HGF.

## Results

### MiR-7 affects genes involved in the cell cycle, cellular movement, cellular assembly and organization pathways

**Figure 1:**
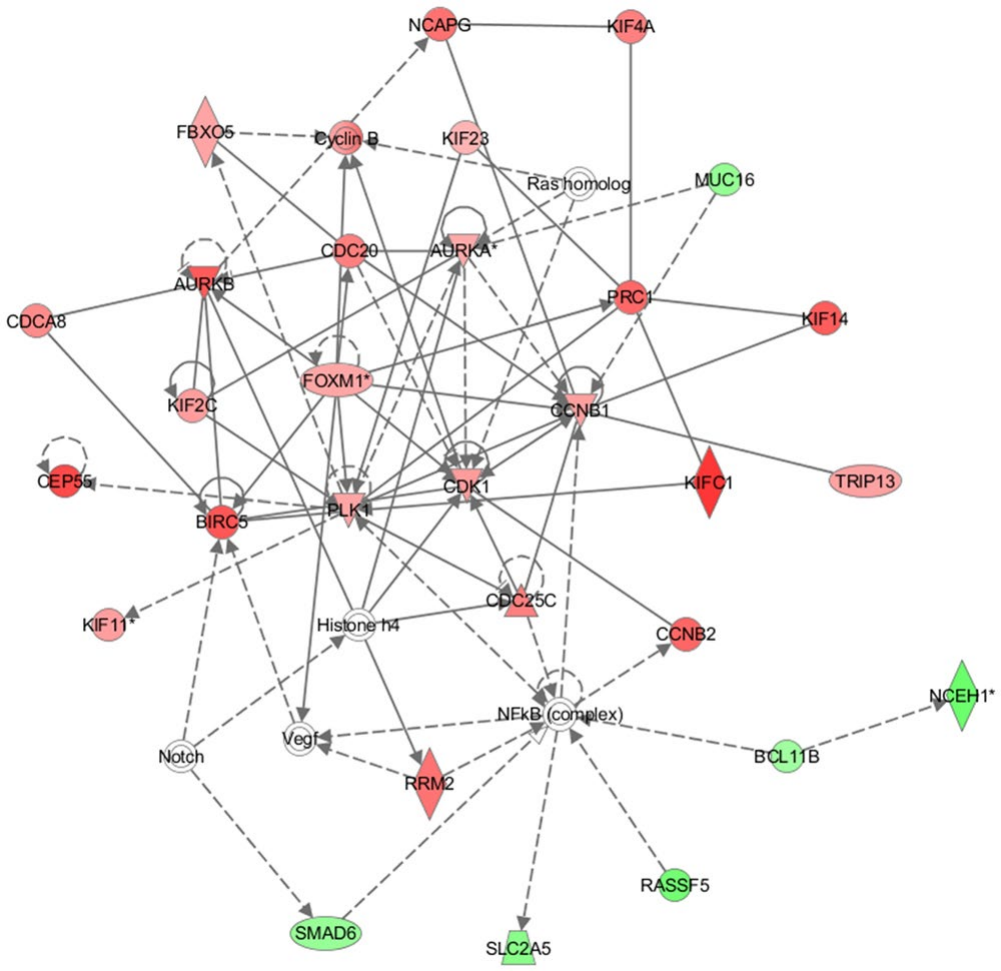
The highest confidence network of genes significantly dysregulated by miR-7. The Ingenuity Pathway Analysis was carried out with the 343 genes that showed significant expression alteration by miR-7 in the MCF-10A cells. The top network identified is “Cell Cycle, Cellular Assembly and Organization, DNA Replication, Recombination, and Repair”. Shapes of red and green color represent upregulated and downregulated genes, respectively. Solid and dashed lines represent direct and indirect interactions, respectively.

**Figure 2:**
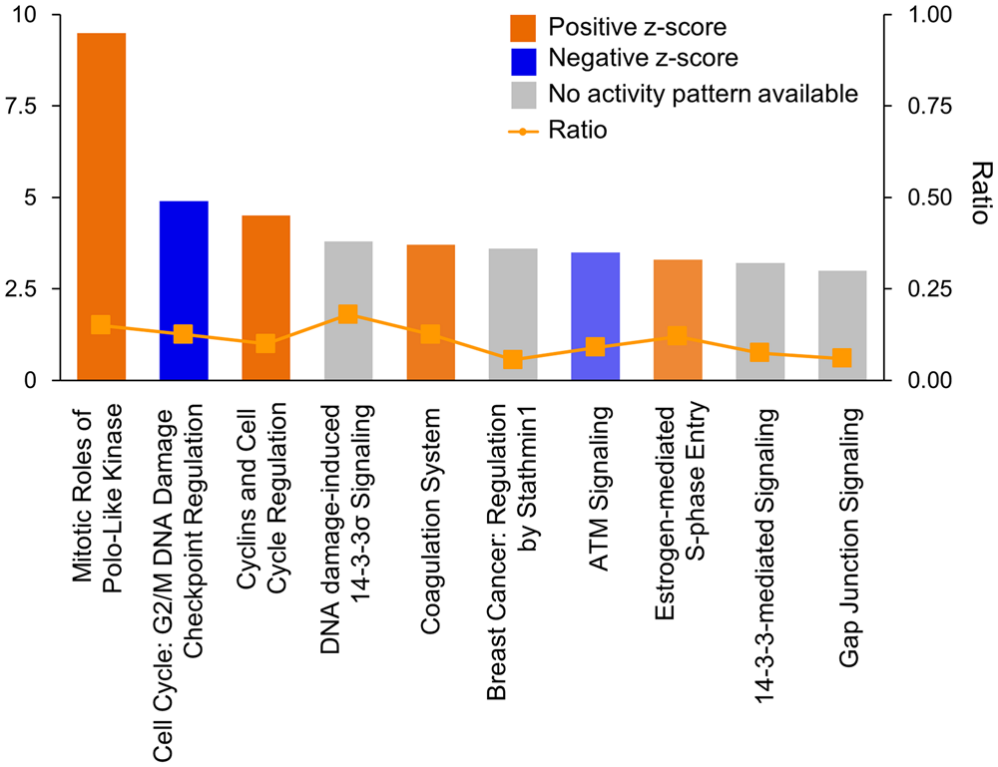
Top 10 canonical pathways of the genes significantly dysregulated by miR-7. The most significant canonical pathway is “Mitotic Roles of Polo-Like Kinase”. Pathways with positive and negative z-scores indicate that the pathways are activated and inhibited, respectively. Ratio is calculated as the number of genes that overlap with the corresponding pathway.

The tumor suppressive activities of miR-7 act through specific target genes, a few of which have been identified thus far. To comprehensively understand the regulatory mechanism through which miR-7 functions in breast tissues, we performed a genome-wide expression analysis in a mammary epithelial cell line, MCF-10A, after overexpressing miR-7 using a mimic miR. Among the 47000 probes on the Illumina Expression BeadChip, 343 genes satisfied our criteria with *P* < 0.05 and |fold change| ≥2 compared to the control miR-transfected cells. An Ingenuity Pathway Analysis was conducted on the gene set, which resulted in the “cell cycle, cellular movement, cellular assembly and organization” network with the highest confidence (Fig. 1 and Table 1). Reassuringly, many genes that were previously identified as miR-7 targets appeared in the network, with an expression coincidence as previously claimed. In detail, MUC16, SLC2A5, SMAD6 and NCEH1 were downregulated by miR-7 and are known to possess oncogenic activities in breast cancer^21,22^, as well as a few other cancers^23^. Notably, “Mitotic roles of polo-like kinase (PLK)” was identified as the most significant canonical pathway (Fig. 2), which represents a highly conserved family of several serine/threonine kinases that regulate cell division, and are often overexpressed in tumors of various cancers^24^. In support of this, genes such as PAK1, FBXO5, CDK1 and KIF23, which directly or indirectly interact with PLK to regulate cell division, were observed to be dysregulated in the network (Fig. 1)^25^. In addition to the IPA, KEGG was also used for the pathway analysis, which identified “cell cycle”-related pathways as the top ones as like as the IPA (Supplementary Fig. S1).

**Table 1:** Genes in the top IPA network showing altered expression in MCF-10A cells due to miR-7.

**Gene symbol**	**Accession no**.	**Description**	**Expression fold change** ^**a**^
KIFC1	NM_002263.2	kinesin family member C1	5.478773
CEP55	NM_018131.3	centrosomal protein 55	4.961733
BIRC5	NM_001168.2	baculoviral IAP repeat containing 5	4.600387
AURKB	NM_004217.2	aurora kinase B	4.471065
KIF14	NM_014875.1	kinesin family member 14	4.438285
CCNB2	NM_004701.2	cyclin B2	4.161032
PRC1	NM_199413.1	protein regulator of cytokinesis 1	4.133515
NCAPG	NM_022346.3	non-SMC condensin I complex subunit G	3.857997
RRM2	NM_001034.1	ribonucleotide reductase regulatory subunit M2	3.80439
KIF4A	NM_012310.3	kinesin family member 4A	3.478862
CDC20	NM_001255.2	cell division cycle 20	3.475324
CDC25C	NM_022809.2	cell division cycle 25C	3.46151
CDCA8	NM_018101.2	cell division cycle associated 8	3.131205
CDK1	NM_001786.2	cyclin dependent kinase 1	2.689246
KIF2C	NM_006845.2	kinesin family member 2C	2.67569
KIF11	NM_004523.2	kinesin family member 11	2.620285
CCNB1	NM_031966.2	cyclin B1	2.587337
TRIP13	NM_004237.2	thyroid hormone receptor interactor 13	2.548576
FBXO5	NM_012177.2	F-box protein 5	2.448304
PLK1	NM_005030.3	polo like kinase 1	2.413278
FOXM1	NM_021953.2	forkhead box M1	2.409883
AURKA	NM_198436.1	aurora kinase A	2.226352
KIF23	NM_004856.4	kinesin family member 23	2.022938
KIFC1	NM_002263.2	kinesin family member C1	5.478773
CEP55	NM_018131.3	centrosomal protein 55	4.961733
BIRC5	NM_001168.2	baculoviral IAP repeat containing 5	4.600387
AURKB	NM_004217.2	aurora kinase B	4.471065
KIF14	NM_014875.1	kinesin family member 14	4.438285
CCNB2	NM_004701.2	cyclin B2	4.161032
PRC1	NM_199413.1	protein regulator of cytokinesis 1	4.133515
BCL11B	NM_138576.2	B cell leukemia/lymphoma 11B	−2.06768
SMAD6	NM_005585.3	SMAD family member 6	−2.25726
MUC16	NM_024690.2	mucin 16, cell surface associated	−2.31952
SLC2A5	NM_003039.1	solute carrier family 2 member 5	−2.56213
RASSF5	NM_182664.2	Ras association domain family member 5	−3.02865
NCEH1	NM_020792.3	neutral cholesterol ester hydrolase 1	−3.19179

^a^The values are obtained by dividing the expression level in miR-7-overexpressed MCF-10A by that in MCF-10A.

### HGF acts as an upstream regulator of miR-7

In order to investigate how miR-7 is modulated, we identified upstream regulators of the miR by performing an IPA’s upstream regulator/mediator analysis of the 343 deregulated genes in the pool. Among 20 putative regulators, HGF had the highest activation z-score (Table 2), with 32 genes affected when HGF was activated or inactivated (Supplementary Table S1). For example, SERPINE1, which was downregulated by miR-7 (2.5-fold decrease), is known to suppress HGF by activating the cleavage of HGF^26^. For the upregulated genes, TPX2 (2.8-fold increase) plays a critical role in the chromosome segregation machinery during mitosis and suppresses tumor cell growth^27^.

**Table 2:** Potential upstream regulators or mediators of miR-7 predicted by target gene analysis.

**Upstream regulator**	**Molecular type**	**Predicted activation state** ^**a**^	**Activation z-score**	***P-value*** **of overlap**
HGF	growth factor	Activated	4.953	2.25E-16
RABL6	other	Activated	4.796	4.91E-25
CSF2	cytokine	Activated	4.7	9.42E-16
PTGER2	g-protein coupled receptor	Activated	4.459	1.52E-28
Vegf	group	Activated	4.31	1.96E-14
RARA	ligand-dependent nuclear receptor	Activated	4.123	5.77E-08
ESR1	ligand-dependent nuclear receptor	Activated	3.963	8.66E-10
FOXM1	transcription regulator	Activated	3.696	4.82E-19
E2f	group	Activated	3.687	1.86E-16
MITF	transcription regulator	Activated	3.483	4.47E-13
BNIP3L	other	Inhibited	−3.317	8.12E-10
CDKN1A	kinase	Inhibited	−3.519	2.07E-33
Irgm1	other	Inhibited	−3.592	1.24E-14
KDM5B	transcription regulator	Inhibited	−3.601	3.74E-08
let-7	microrna	Inhibited	−3.734	4.12E-15
CDKN2A	transcription regulator	Inhibited	−3.764	3.21E-11
phorbol myristate acetate	chemical drug	Inhibited	−4.448	2.89E-07
TP53	transcription regulator	Inhibited	−4.529	5.91E-26
NUPR1	transcription regulator	Inhibited	−5.488	6.02E-13
calcitriol	chemical drug	Inhibited	−5.68	1.86E-25

^a^The status of the regulator that results in the same expression profile of the target genes of miR-7 in the miR-7-overexpressing MCF-10A.

**Figure 3:**
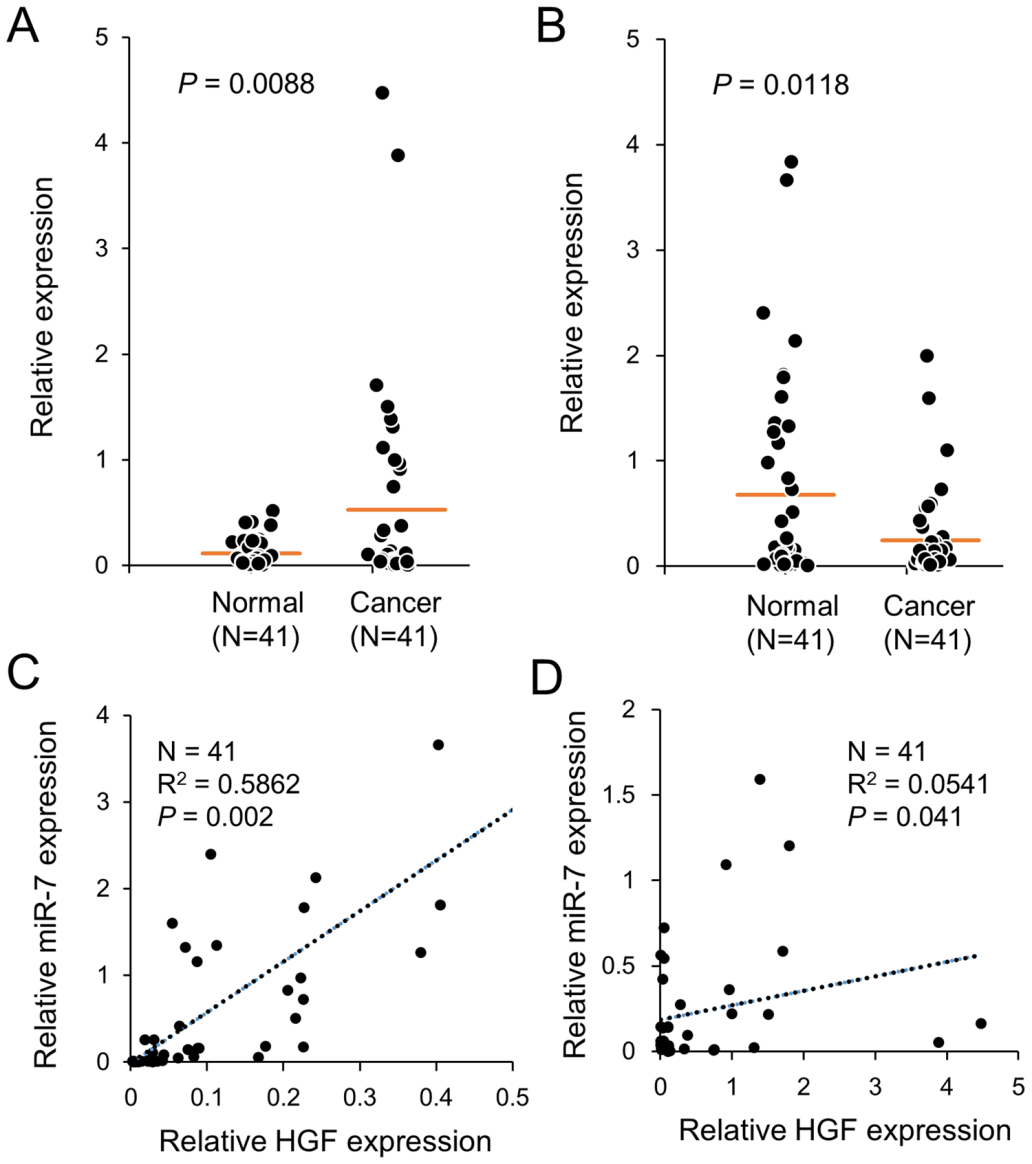
Expression of HGF and miR-7 is strongly associated in normal breast tissue. Expression of HGF (**A**) and miR-7 (**B**) was examined by qPCR in 41 pairs of breast cancer tissues and nearby normal tissues and represented by dot plots. Association between HGF and miR-7 was determined in breast normal (**C**) and cancer tissues (**D**) by linear regression, and represented by the coefficient of determination (*R* ^2^).

The regulatory relationship between miR-7 and HGF was also investigated by examining the expression of HGF and miR-7 in 41 pairs of breast cancer and normal tissues. HGF showed an increased expression in cancer tissues compared to normal tissues, while miR-7 showed the opposite pattern, confirming the observations in previous studies (Fig. 3A,B). Notably, comparing the expression between the two genes revealed a high positive association in the normal tissues (*R* ^2^ = 0.58, *P* < 0.01) but not in the cancer tissues (*R* ^2^ = 0.05, *P* < 0.05) (Fig. 3C,D). When examined in a few other tissues including liver, head and neck, colon, and bladder, no remarkable association was found regardless normal or caner tissue (Supplementary Fig. S2). These results imply that the association of HGF and miR-7 expression is stronger in normal tissues than in cancer tissues for breast and further that it is not a general phenomenon through body tissues.

### HGF controls expression of MiR-7 and its target genes

**Figure 4:**
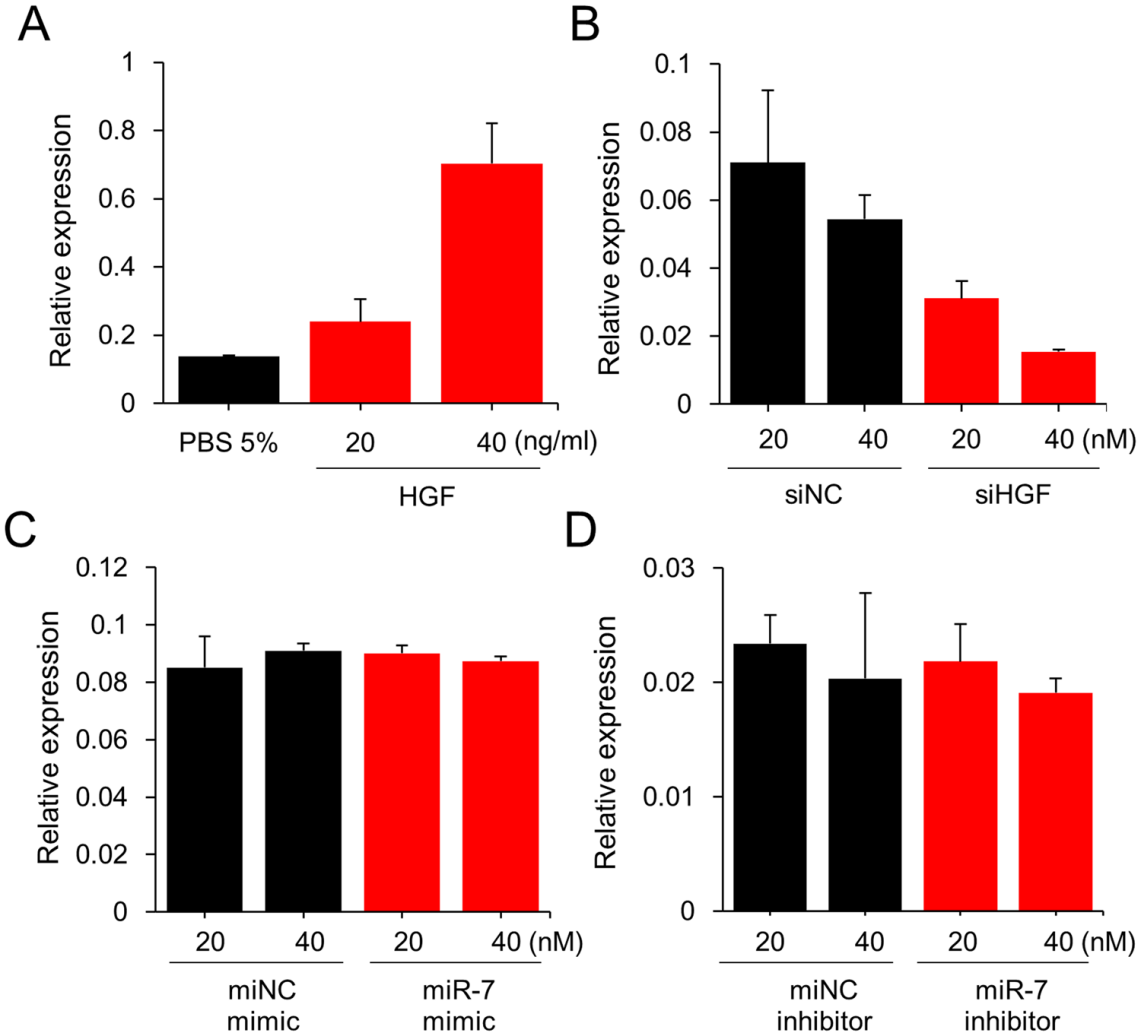
HGF upregulates miR-7 in MCF-10A cells. (**A**) Cells were treated with HGF for 24 hours, after which the expression level of miR-7 was measured by qPCR. (**B**) HGF expression was downregulated with siRNA (siHGF) and the expression level of miR-7 was measured by qPCR 24 hours after transfection. siNC, control siRNA. (**C** and **D**) miR-7 was overexpressed using mimic miR or downregulated using an inhibitor miR. Twenty-four hours after treatment, the expression level of HGF was measured by qPCR. The expression levels of HGF and miR were normalized to GAPDH and U6 RNA, respectively. All experiments were performed in triplicate and indicated as mean ± SE. siNC, siRNA negative control; miNC, microRNA negative control.

Based on the results that HGF and miR-7 share many target genes in common and their expression is strongly associated in normal breast tissue, we examined the effects of HGF in a normal breast cell line, MCF-10A. First, the effect of HGF and miR-7 on cell proliferation was examined. As results, HGF alone increased the growth rate of the cell, while miR-7 mimic alone decreased the growth rate. When miR-7 mimic was co-treated with HGF, it deteriorated the growth-stimulation effect of HGF (Supplementary Fig. S3). Next, we investigated the influence of HGF on the expression of miR-7 as well as its target genes such as *FAK, PAK1, EGFR, KLF4* and *SET8*, which were all validated in our microarray assay, as well as in previous studies^5,28–31^. When MCF-10A cells were treated with 20 or 40 ng/ml HGF, a resulting upregulation of miR-7 was observed (Fig. 4A); on the other hand, a downregulation of miR-7 was also observed when cells were treated with siRNA against HGF (Fig. 4B and Supplementary Fig. S4A). Both occasions showed a dose-dependent response.

To investigate whether the regulation of miR-7 by HGF functions in a feedback loop, we performed a pulse-chase experiment to check for HGF expression after miR-7 was either upregulated through mimic miR or downregulated through an inhibitor miR (Supplementary Fig. S4B,C). In both cases, no significant expression changes in HGF was observed, implying that there is no regulation of HGF by miR-7 (Fig. 4C,D).

**Figure 5:**
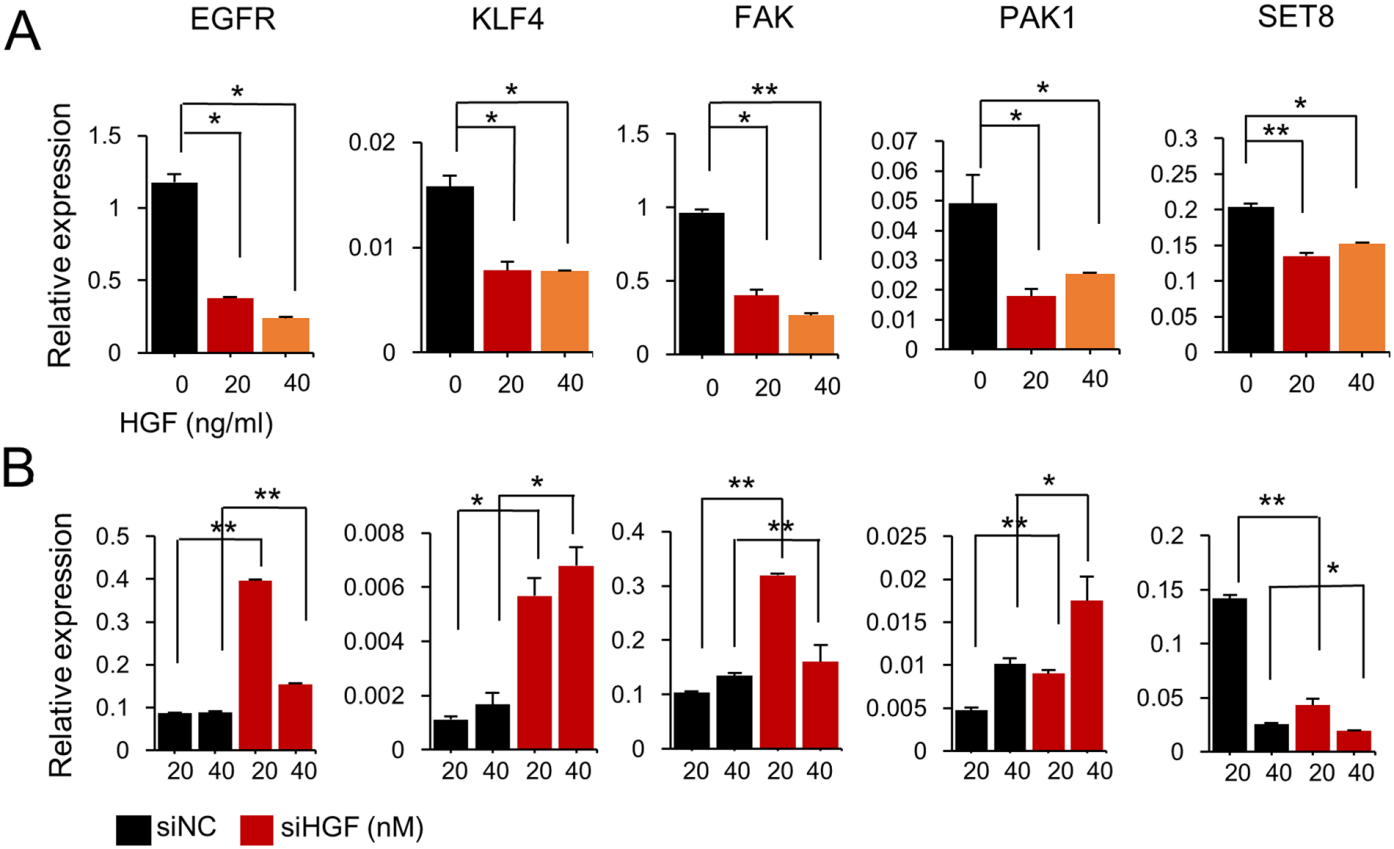
HGF regulates target genes of miR-7. The expression of selected target genes for miR-7 was examined after treatment of the MCF-10A cells with HGF protein (**A**) and siRNA (**B**) to induce upregulation or downregulation of HGF, respectively. Twenty-four hours after treatment, the expression level of target genes was measured by qPCR. All the target genes were downregulated by HGF but upregulated by siRNA against HGF, except for SET8. All experiments were performed in triplicates and indicated as mean ± SE. *P < 0.05; **P < 0.001.

Five genes that are known to be direct targets of miR-7 were then selected, and the effect of HGF on their expression was monitored by qPCR. Treatment of HGF to MCF-10A cells resulted in a downregulation in all genes (Fig. 5A). On the other hand, when HGF was inhibited by siRNA, an upregulation was observed in the expression of all genes except for SET8 (Fig. 5B). Taken together, these results suggest that HGF downregulates cell proliferation-related genes by upregulating miR-7, which acts to suppress the cancer progression of normal breast cells.

## Discussion

This study aimed to explore upstream regulators of miR-7, a microRNA that has shown tumor suppressor functions in many cancers including breast cancer. To accomplish this, we adopted a strategy wherein potential upstream regulators or mediators of miR-7 were identified by screening for commonly affected genes by miR-7 and its regulators. The potential regulators appeared in diverse subcellular locations, i.e., ligands such as HGF and VEGF, receptors on the cell membrane such as PTGER2, signaling kinases such as CDKN1A and CDKN2A, and transcription factors such as TP53 and MITF.

One remarkable characteristic of the identified potential regulators is sharing of the PI3K/AKT, MAPK, or JNK in common *en route* the signaling pathway. Especially, 13 genes including VEGF^32^, RARA^33^ and ESR1^34^ activate the signaling pathway by regulating MAPK. A previous study also identified TGF-β signaling to be responsible for miR-7 inhibition in the MCF-7 breast cancer cell line^35^. The regulatory pathways from ligands and/or receptors therefore become complicated, and the detailed pathway from HGF to miR-7 should be identified in a future study. A few of the potential mediators of miR-7 have already been known to affect or be affected by miR-7. MiR-7 was shown to be regulated by estrogen and to target signaling intermediates such as EGFR, IGF1R and IRS-2^36^. KLF4, a direct target of miR-7, acts on the VEGF promoter to induce its mRNA and protein levels. This miR-7-KLF4-VEGF signaling axis contributes to the regulation of angiogenesis in human umbilical vein endothelial cells^37^. Upregulation of miR-7 targeted genes implicated in the TP53 pathway, such as Ak1 and p21, also led to the controlled growth of cortical neural progenitors^38^.

HGF triggers multiple signaling pathways, including the conventional PI3K/AKT and MAPK pathways, coupled with the Hic-5-reactive oxygen species (ROS)-c-jun-N-terminal kinase (JNK) cascade^39^. These signaling events eventually increase the expression of a group of genes such as N-cadherin, vimentin and Zeb1, which trigger metastatic changes including epithelial mesenchymal transition (EMT), enhancement of motility and the invasiveness of tumor cells^40^. The majority of target genes activated by HGF have pro-proliferation activities, while those suppressed by HGF have anti-proliferation activities. HGF, therefore, has been generally considered as an oncogenic growth factor. HGF also has specific roles associated with cell proliferation in normal cells, even though these are less well-known than its oncogenic roles. Previous studies indicate that HGF increased mammary epithelial cell proliferation by acting through the PI3K-including mitogenic pathway, which thus induced a tubulo-ductal morphological response^41^.

In contrast to previous studies, the four selected oncogenes, EGFR, KLF4, FAK and PAK1^42–45^ were downregulated by HGF in MCF-10A cells. Because our result suggest that the downregulation of the genes are mediated via miR-7, we speculate that HGF acts as a double-edged sword depending on cellular status or cell type. This idea is supported by the upregulation of miR-7 in the HGF-treated MCF-10A cells, and by the strong association between miR-7 and HGF expression in normal mammary tissues. There have also been previous studies indicating the tumor-suppressive activity of HGF by abrogating the oncogenic effects of c-Myc during early stages of liver carcinogenesis^46^, as well as enhancing the differentiation activity in mammary glands^14^ and hepatocytes^13^. Therefore, during tumorigenesis, upregulated HGF would drive the expression of oncogenes, while in normal cells, miR-7 would mediate HGF to downregulate the same oncogenes. Recently, effective computational models such as PBMDA^47^, HGIMDA^48^, and RKNNMDA^49^ have been constructed to identify disease-related miRNA biomarkers. These bioinformatics-based approaches should help to gain further insight into the molecular mechanisms of HGF and miR-7. In addition, dynamic feedback modeling^50^ and Cancer Hallmark Network Framework^51^ could give us an insight to better understand the feedback loop of miR-7 in breast cancer.

Our genome-wide analysis revealed that a set of oncogenes was downregulated by miR-7, while many tumor suppressors were upregulated, supporting the tumor suppressive activity of miR-7. In addition, a group of genes that was not previously identified as targets of miR-7 was discovered to rank at the top of the network. For example, FAM83A (2.4-fold decrease) is an oncogene that activates CRAF/MAPK signaling and drives epithelial transformation^52^, while MUC16 (2.3-fold decrease) is a tumor marker that induces breast cancer cell proliferation by interacting with JAK2^21^. Whether these genes are directly regulated by miR-7, however, should be elucidated in a further study.

SET8, being different from other target genes of miR-7, was downregulated regardless of HGF overexpression or inhibition. This observation suggests the existence of other regulatory pathways in between HGF and SET8, which are independent of miR-7. SET8 is the sole protein lysine methyltransferase to monomethylate histone 4 lysine 20 (H4K20) and its function has been implicated in normal cell cycle progression and cancer metastasis^53^. Recently, a study revealed that miR-502 directly targets SET8 to suppress cell proliferation and cell cycle^54^.

In conclusion, HGF was identified as an upstream regulator of miR-7 due to their sharing of a group of target genes that showed similar gene expression changes. These genes include oncogenes such as EGRF, KLF4, FAK and PAK1, which were downregulated by either HGF or miR-7. In addition, there seems to be no feedback regulation of HGF by miR-7. Because miR-7 acts as a tumor suppressor, the HGF/miR-7 pathway could potentially explain the tumor-suppressive effects of HGF in normal cells.

## Materials and Methods

### Cell culture and transfection

The normal epithelial breast cell line MCF-10A was purchased from the American Type Culture Collection (ATCC; Manassas, VA, USA) and cultured in MEBM basal medium (Lonza, Basel, Switzerland) supplemented with the MEGM Single Quot Kit (Lonza) and cholera toxin (List Biological Labs, Campbell, CA) under a humid environment with 5% CO_2_ at 37 °C. MiR-7-5p mimic, control miR (miNC), miR-7-5p inhibitor, control inhibitor (miNC inhibitor), siHGF and siRNA control (siNC) were synthesized by Bioneer (Korea). All miRs, inhibitors and siRNAs were diluted in OptiMEM I Medium (Gibco, Los Angeles, CA, USA) and transiently transfected into cells at a final concentrations of 20 and 40 nM using Lipofectamine RNAiMAX (Invitrogen, Carlsbad, CA, USA).

### Study subjects

Forty-one breast cancer tissues were obtained from patients who underwent surgery between 2013 and 2014 at the National Cancer Center (NCC) in Korea. All patients provided written informed consent to donate removed tissue to NCC, and samples were obtained according to protocols approved by the Research Ethics Board of NCC.

### RNA extraction and real-time qPCR

Total RNA was harvested from miR- or siRNA-transfected cells and breast tissues using the ZR-Duet DNA/RNA MiniPrep kit (Zymo research, Irvine, CA, USA) according to the manufacturer’s recommendations. In order to quantify the levels of mature miR-7, the extracted RNA was reverse transcribed to cDNA using the miScript II RT Kit (Qiagen, Valencia, CA, USA). Afterwards, quantitative RT-PCR (qPCR) was performed with the miScript SYBR Green PCR Kit (Qiagen) and miScript Primer Assays as the primers. For the quantification of protein coding gene’s expression level, reverse transcription was carried out with ReverTra Ace qPCR RT Master Mix with gDNA remover (Toyobo, Japan) and PCR was performed with Kapa SYBR Fast qPCR Kit Master Mix ABI Prism (Kapa Biosystems, Inc., Wilmington, MA, USA). The reactions were assayed in triplicate on an ABI 7300 instrument (Applied Biosystems, Foster City, CA, USA). The expression of miR-7 and protein coding genes was normalized using endogenous U6 and GAPDH with the 2^−ΔΔCt^ calculation, respectively. The primers used for amplification of miRs and coding genes are listed in Supplementary Table S2.

### HGF treatment

3 × 10^3^ MCF-10A cells in culture media were seeded in each well of a 96-well plate, and were treated with HGF dissolved in PBS to a final concentrations of 20 and 40 ng/ml. As a control, PBS was used to a final concentration of 5%. The cells were cultured for 24 hours and total RNA was isolated for the expression analysis of miR-7 and coding genes. To verify the cell activation by HGF, the cell growth rate was measured using Cell Counting Kit-8 (Dojindo, Japan) at 450 and 600 nm 2 h after incubation with 10 μl of CCK-8 reagent to a well. The absorption value at 450 nm was subtracted from the value at 600 nm for turbidity removal.

### Expression microarrays and pathway analysis

One microgram of total RNA from miR-7- or miNC-transfected MCF-10A cells was used for the expression microarray of Illumina Human HT-12 v4 Expression BeadChip (Illumina, San Diego, CA). Among 47000 probes on the chip, probes with detection *P*-value < 0.05 and |fold change| ≥ 2 were screened as significantly deregulated genes. Relevant networks and canonical pathways were generated using the Ingenuity Pathway Analysis (IPA) (Qiagen). The KEGG pathway enrichment analysis was performed by the KEGG Orthology Based Annotation System (KOBAS)^55^. The expression microarray data were deposited into the Gene Expression Omnibus database (http://www.ncbi.nlm.nih.gov/geo/) with the series accession number GSE102758. The mining of upstream regulators of miR-7 was carried out on the IPA platform which analyzed commonly regulated genes by HGF and miR-7.

### Statistical analysis

Gene expression data were represented as the mean ± standard error of three independent experiments and analyzed by Student’s *t*-test using SPSS for Windows, version 17.0 (SPSS, Chicago, IL, USA). Differences were considered statistically significant when the *P*-value is lower than 0.05. Linear regression was conducted to calculate the coefficient of determination (*R* ^2^) and the statistical significance of the correlation between miR-7 and HGF expression.

## Electronic supplementary material

Supplementary Information
