# Supplementary material for: MicroRNA-7-5p mediates the signaling of hepatocyte growth factor to suppress oncogenes in the MCF-10A mammary epithelial cell
Source: Sci Rep. 2017 Nov 13;7:15425. doi: 10.1038/s41598-017-15846-z (PMC5684415; doi:10.1038/s41598-017-15846-z)

MicroRNA-7-5p mediates the signaling of hepatocyte growth factor to suppress oncogenes in the MCF-10A mammary epithelial cell

Dawoon Jeong, Juyeon Ham, Sungbin Park, Seungyeon Lee, Hyunkyung Lee, Han-Sung Kang, and Sun Jung Kim

Supporting Information

Table S1. Genes that are deregulated by miR-7 and predicted to be affected by HGF

Gene symbol	Description	Prediction[a]	Expression fold change
UBE2C	Ubiquitin-Conjugating Enzyme E2C	Activated	5.17
TTK	TTK Protein Kinase	Activated	2.785
TRIP13	Thyroid Hormone Receptor Interactor 13	Activated	2.549
TPX2	TPX2, Microtubule-Associated	Activated	2.849
STIL	SCL/TAL1 Interrupting Locus	Activated	2.211
PTGS1	Prostaglandin-Endoperoxide Synthase 1	Activated	-2.067
PRC1	Protein Regulator Of Cytokinesis 1	Activated	4.134
PLK1	Polo-Like Kinase 1	Activated	2.413
NEK2	NIMA-Related Kinase 2	Activated	4.744
NDC80	NDC80 Kinetochore Complex Component	Activated	2.189
MELK	Maternal Embryonic Leucine Zipper Kinase	Activated	2.8
KIF2C	Kinesin Family Member 2C	Activated	2.676
KIF20B	Kinesin Family Member 20B	Activated	2.074
KIF11	Kinesin Family Member 11	Activated	2.62
ISG20	Interferon Stimulated Exonuclease Gene	Activated	2.182
HMMR	Hyaluronan-Mediated Motility Receptor	Activated	5.12
FOXM1	Forkhead Box M1	Activated	2.41
CRY1	Cryptochrome Circadian Clock 1	Activated	2.537
CENPF	Centromere Protein F, 350/400kDa	Activated	4.192
CDKN3	Cyclin-Dependent Kinase Inhibitor 3	Activated	2.798
CDK1	Cyclin-Dependent Kinase 1	Activated	2.689
CDC45	Cell Division Cycle 45	Activated	4.183
CDC25C	Cell Division Cycle 25C	Activated	3.462
CDC20	Cell Division Cycle 20	Activated	3.475
CCNF	Cyclin F	Activated	2.209
CCNE2	Cyclin E2	Activated	2.083
BUB1	BUB1 Mitotic Checkpoint Serine/Threonine Kinase	Activated	3.17
BIRC5	Baculoviral IAP Repeat Containing 5	Activated	4.6
AURKB	Aurora Kinase B	Activated	4.471
AURKA	Aurora Kinase A	Activated	2.33
CRYAB	Crystallin, Alpha B	Inhibited	-2.355
BNC1	Basonuclin 1	Inhibited	-2.055

[a]The status of HGF that results in the same expression profile of the target gene of miR-7 in the miR-7-overexpressing MCF-10A

Table S2. Sequences of primers applied in this study

Genes	Product name or sequence (5'-3')	Supplier	Catalog no.
miR-7-5p	Hs_miR-7_2 miScript Primer Assay	Qiagen	MS00032116
RNU6	Hs_RNU6-2_11 miScript Primer Assay		MS00033740
GAPDH	F: CAGGAGGCATTGCTGATGAT	Genotech	
	R: GAAGGCTGGGGCTCATTT		
HGF	F: CGTCATATCTTCTGGGAACCA		
	R: TGTGGTATCACCTTCACAACG		
EGFR	F: GCGTTCGGCACGGTGTATAA		
	R: GGCTTTCGGAGATGTTGCTTC		
KLF4	F: GTCTTGAGGAAGTGCTGAGC		
	R: ATCGTCTTCCCCTCTTTGGC		
SET8	F: ACTTACGGATTTCTACCCTGTC		
	R: CGATGAGGTCAATCTTCATTCC		
FAK(PTK2)	F: ACCAGAGGAGTGGAAATATGAA		
	R: AATCGCTCTTCACCTGTTGAT		
PAK1	F: AGGTGGCCATTAAGCAGATG		
	R: CTCCCACGAGGTAACTGTCC		

Figure S1. Pathways obtained from KEGG enrichment analysis of genes differentially expressed by miR-7. The KEGG pathways were ranged according to their transcriptional coverage the *P*-value. The top six pathways with the *P*-value <0.05 were presented.

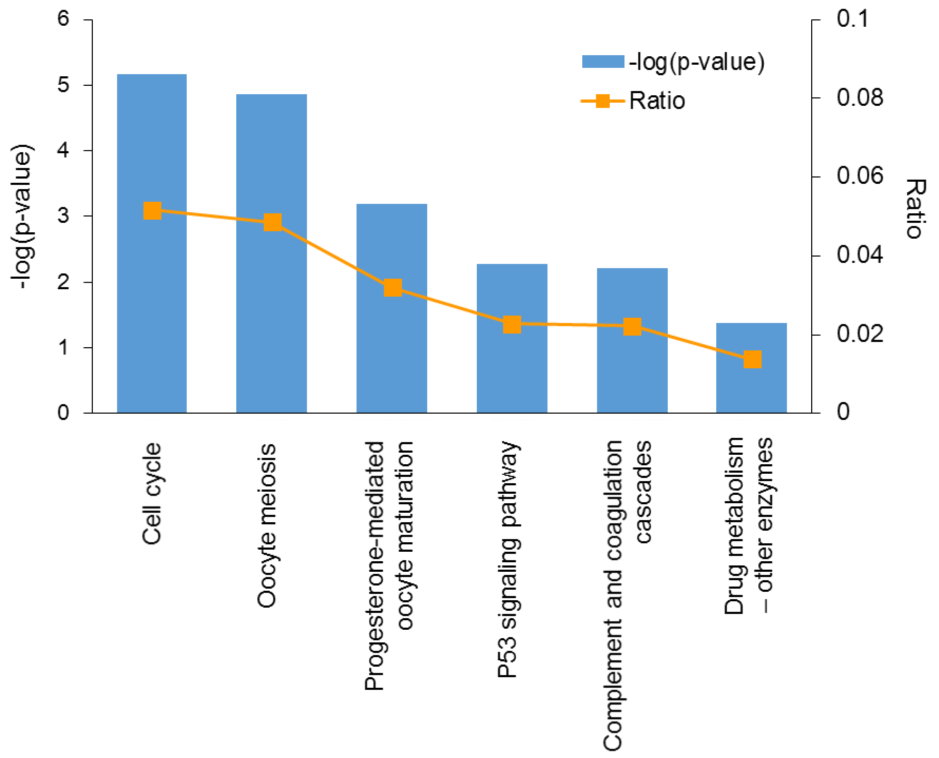

Figure S2. Association of HGF and miR-7 expression in human tissues. Expression level of HGF and miR-7 in various tissues was extracted from the TCGA database (https://cancergenome.nih.gov/) and the association analysis was performed on the MethHC platform (http://methhc.mbc.nctu.edu.tw/php/index.php). Only tissues with the number > 40 were considered for analysis. The association between HGF and miR-7 was determined by linear regression, and represented by the coefficient of determination (R^2). A, normal liver; B, liver cancer; C, normal head and neck; D, head and neck cancer; E, bladder cancer; F, colon cancer.

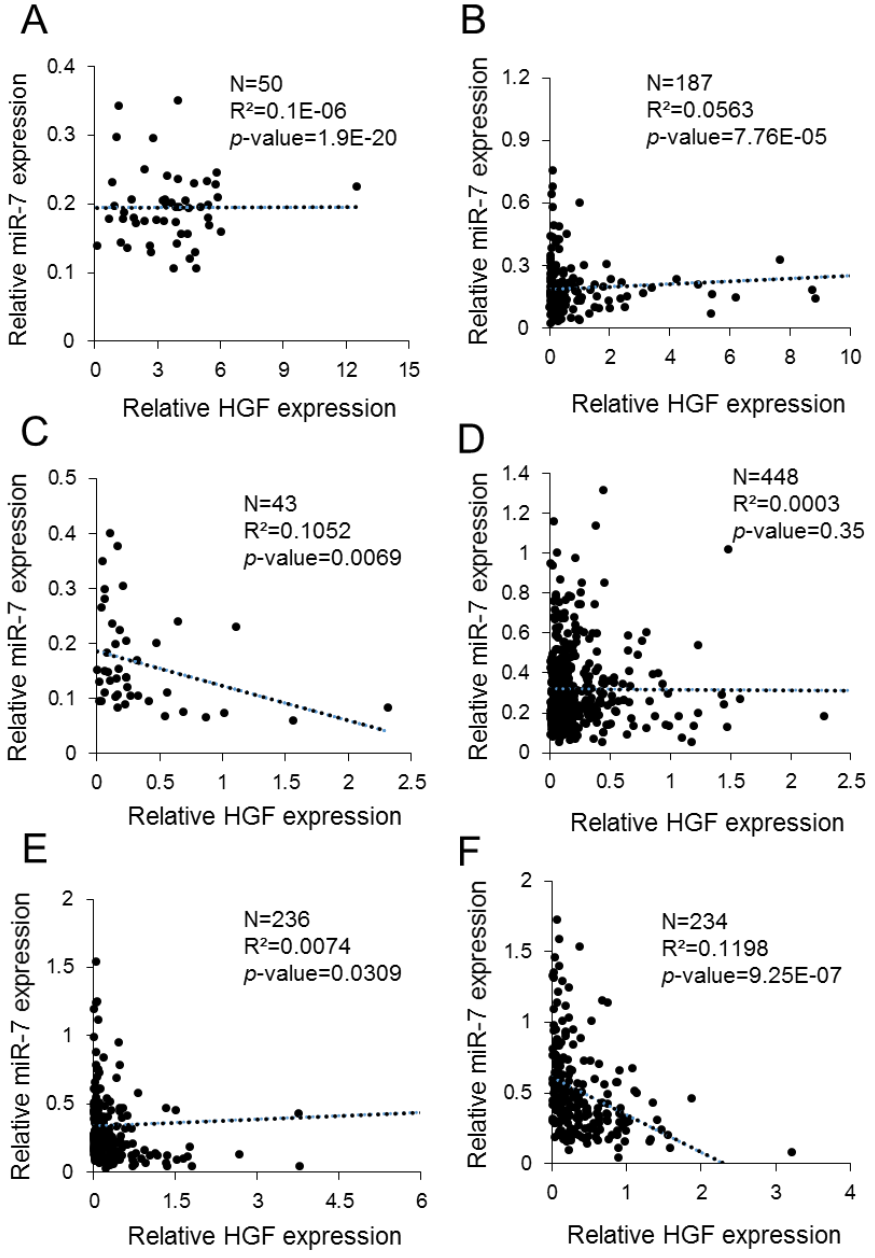

Figure S3. Effect of HGF and miR-7 on the cell proliferation of MCF-10A. The cells were treated with HGF (A), miR-7 (B), or both HGF and miR-7 (C). HGF protein was added into the culture media and miR-7 mimic (or inhibitor) was transiently transfected. In B and C, 40 ng/ml of HGF, 40 nM of siHGF, and 20 nM of mimic and inhibitor for miR were used. At the indicated time or day, the proliferation was monitored by measuring the CCK-8 dye-based absorbance. All experiment were carried out in triplicate and indicated as mean ± SE. NC, negative control; siHGF, siRNA for HGF.

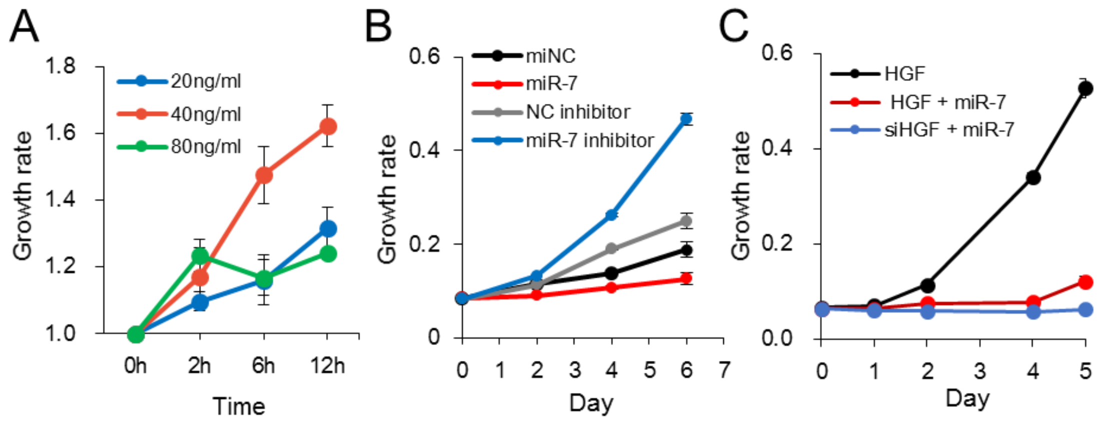

Figure S4. Confirmation of treatment of mimic miR and siRNAs onto MCF-10A cells. (A) Appropriate treatment of mimic miR and siRNA to the cell culture was confirmed by qPCR. (A) Downregulation of HGF by an siRNA. (B) Upregulation of miR-7 by a mimic miR. (C) Downregulation of miR-7 by an inhibitor. All experiment were carried out in triplicate and indicated as mean ± SE. NC, negative control.

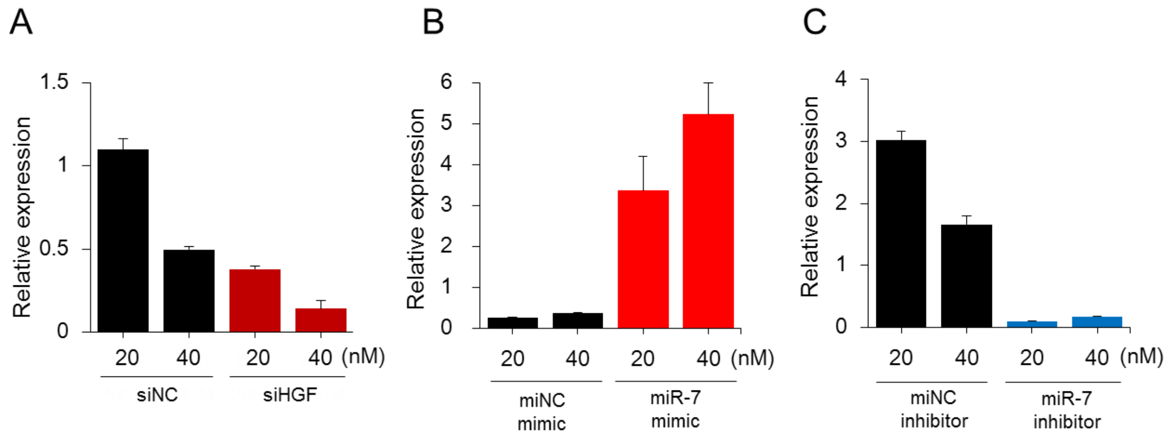

Supplement: Supplementary file 1 — Supplementary Information [file 41598_2017_15846_MOESM1_ESM.pdf]
